# Small RNA Diversity in Plants and its Impact in Development

**DOI:** 10.2174/138920210790217918

**Published:** 2010-03

**Authors:** Christine Lelandais-Brière, Céline Sorin, Marie Declerck, Abdelali Benslimane, Martin Crespi, Caroline Hartmann

**Affiliations:** 1 Institut des Sciences du Végétal, Centre National de la Recherche Scientifique (C.N.R.S.), F-91198 Gif-sur-Yvette Cedex, France; 2 Université Paris Diderot, 5 rue Thomas Mann 75205 Paris Cedex 13, France; 3 Département de Biologie, Faculté des Sciences Semlalia, Université Cadi Ayyad, BP S15, 40001 Marrakech, Morocco

## Abstract

MicroRNAs are a class of non-coding RNAs involved in post-transcriptional control of gene expression, either *via* degradation or translational inhibition of target mRNAs. Both experimental and computational approaches have been used to identify miRNAs and their target genes. In plants, deep sequencing methods have recently allowed the analysis of small RNA diversity in different species and/or mutants. Most sequencing efforts have been concentrated on the identification of miRNAs and their mRNA targets have been predicted based on complementarity criteria. The recent demonstration that certain plant miRNAs could act partly *via* inhibition of protein translation certainly opens new fields of analysis for plant miRNA function on a broader group of targets. The roles of conserved miRNAs on target mRNA stability have been analysed in different species and defined common mechanisms in development and stress responses. In contrast, much less is known about expression patterns or functions of non-conserved miRNAs. In this review, we focus on the comparative analyses of plant small RNA diversity and the action of si/miRNAs in post-transcriptional regulation of some key genes involved in root development.

## 1. INTRODUCTION

This last decade, one of the most important revolutions in biology is the identification and characterization of the roles of non-coding RNAs and, more precisely the small RNAs, on the regulation of gene expression. In plants, there are two families of small RNAs that repress gene expression at transcriptional or post-transcriptional levels: short interfering RNA (siRNAs) and microRNAs (miRNAs). The discovery of the small RNAs has strongly modified our view of gene regulation and developmental control in plants and animals. Indeed, plant miRNAs play a critical role in nearly all biological processes: development, differentiation, biotic and abiotic stress responses. Endogenous siRNAs may also have regulatory functions in processes such as maintenance of genomic integrity, developmental patterning and response to environmental stresses. Moreover, these discoveries may provide new tools for the regulation of gene expression in eukaryotes.

In this review, we will discuss recent results on the analysis of small RNA diversity and their mode of action in plants and we focus on the action of specific miRNAs in the control of shoot organogenesis and root growth and development.

## 2. SMALL RNAs BIOGENESIS AND mRNA TARGET REGULATION

The origin of endogenous small RNAs differs as siRNAs are generated from long intermolecular double strand RNAs (dsRNA) whereas miRNAs are produced from intramolecular dsRNAs.

### A. miRNA Biogenesis and Gene Organization

**Fig. (1). Schematic representation of the plant miRNA biogenesis pathway.**
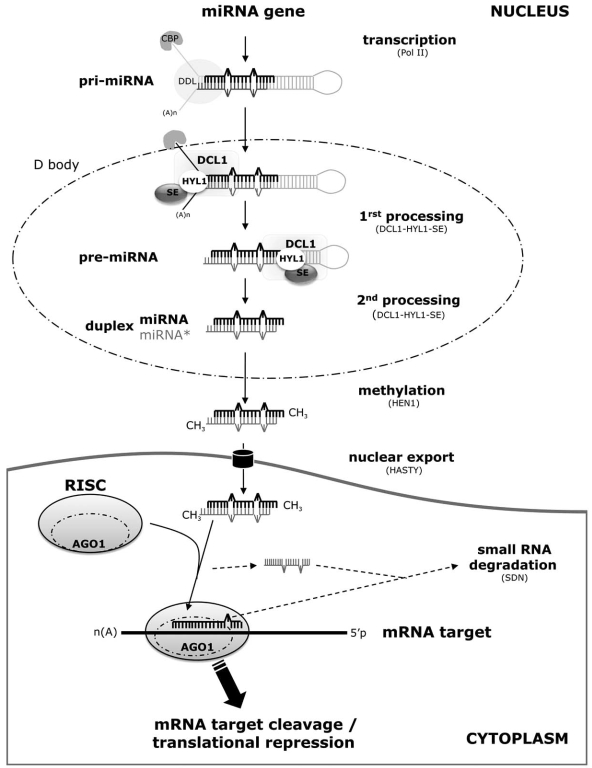
In the nucleus, the miRNA gene is transcribed by RNA Polymerase II (POLII) in a primary transcript (pri-miR). CAP–BINDING PROTEINS (CBP) are required for the first step of miRNA processing [1, 2]. The pri-miR contains an imperfect stem-loop structure. The miRNA precursor (pre-miR) is then excised from the pri-miR [3] through the action of DICER-LIKE 1 (DCL1), associated with other dsRNA-binding proteins such as HYPONASTIC LEAVES 1 (HYL1) and SERRATE (SER). The DAWDLE (DDL) protein, a forkhead-associated domain protein, seems also to be involved in pri-miR excisions [4]. The pre-miR is further processed by the same DCL complex (or D-body) to generate a 21 nt double stranded miRNA (the miRNA/miRNA* duplex) which is methylated on the 3’ ends by the HUA ENHANCER 1 (HEN1) protein. After export of the miRNA/miRNA* duplex out of the nucleus through HASTY, the mature miRNA is preferentially incorporated into the RNA-Induced Silencing Complex (RISC) containing ARGONAUTE 1 (AGO1) [5, 6]. The miR* is released. Inside the RISC complex, the base pairing of the miRNA and its mRNA target(s) leads to the cleavage and/or the translational repression of the target. Recent results suggest that miRNAs could be degraded by the SMALL RNA DEGRADING NUCLEASE 1 (SDN1) protein [13].

In plants, the miRNA biogenesis pathway has been elucidated in *Arabidopsis* by forward genetics (Fig. **1**). MiRNAs are generally transcribed by RNA Polymerase II. Proteins such as CAP – BINDING PROTEINS (CBP) CBP20 and CBP80, normally involved in the stabilization and splicing efficiency of mRNAs, are also required for the first step of miRNA processing [1, 2]. The primary transcriptional products, called primary miRNAs (pri-miRs), contain an imperfect stem-loop structure from which the precursor (pre-miR) is excised through the action of a dsRNA-RNAse called DICER-LIKE 1 (DCL1). This protein associates with other dsRNA-binding proteins such as HYPONASTIC LEAVES 1 (HYL1) and SERRATE (SER) to achieve this processing step [3]. Recently, the involvement of the DAWDLE (DDL) protein, a forkhead-associated domain protein, has also been shown, which probably interacts with DCL1 in primiR excisions. Indeed, the amounts of pre-miRNAs and miRNAs are reduced in *ddl* mutants [4]. The pre-miR is further processed by the same DCL1 protein complex to generate predominantly a 21 nt canonical double stranded miRNA (the miRNA/miRNA* duplex) which is methylated on the 3’ ends by the HUA ENHANCER 1 (HEN1) protein. Then, the miRNA/miRNA* duplex is exported out of the nucleus by HASTY and, once in the cytoplasm, the single miRNA strand is preferentially incorporated into the RNA-Induced Silencing Complex (RISC). This complex contains ARGONAUTE 1 (AGO1), one of the major proteins of miRNA pathway, that is responsible for the cleavage of the target mRNA through base pairing between the miRNA and its target. Thus, the miRNA defines the specificity of action of the RISC complex. High-throughput sequencing of RNAs that immunoprecipitate with specific AGO proteins revealed that, at least in *Arabidopsis*, the basis of small RNA selection by AGO proteins could be the nature of the 5’ terminal nucleotide (nt) [5, 6]. For example, 21 nt small RNAs that begin with a 5’ uridine are selectively incorporated in RISC containing AGO 1 protein [5].

In *Arabidopsis*, 4 *DCL* genes have been identified and shown to release different miRNA sizes (e.g. 21-22 nt for *DCL1* and 23-24 nt for *DCL3*). Studies of *dcl* mutants have clearly shown that at least certain DCL functions may act redundantly [7]. Recently, Vazquez *et al.* [8], by using northern blot hybridizations, found that 41 among 94 *Arabidopsis* miRNA families analysed produced two miRNA populations: the canonical 21 nt miRNA and an additional 23 to 25 nt form called long miRNA. In certain cases, and especially for the non-conserved miRNA families, the long miRNAs were the most abundant form. They showed that these canonical and long miRNAs are processed from the same pre-miR by DCL1 and DCL3 respectively, depending on their expression pattern. Indeed, in flowers, where DCL3 was more expressed (+10 fold higher than in leaves), the long miRNAs appeared more abundant than the 21 nt miRNAs. Hence, the same pre-miRNA can be processed by different DCL enzymes to yield different classes of small RNAs.

Plant miRNA genes are generally found in intergenic regions of the genome and are subdivided into highly, moderately and non-conserved classes in relation to their presence across the plant kingdom [9]. Certain miRNAs are encoded by a single gene, whereas others are encoded by many loci that are sometimes clustered. The miRNA database miRBase (http://microrna.sanger.ac.uk/sequences/) encompasses 21 miRNA families, which are present in four angiosperm species, *Arabidopsis*, poplar, grapevine and rice (Table **1**). For most of these miRNAs, the targets seem also to be conserved, suggesting a common mechanism of regulation [10]. Although only 12 of them were found in other embryophyta, these genes are considered as ancient miRNAs in comparison with young emergent miRNAs, specific of one or few species and often encoded by a single locus. Several non-conserved miRNA genes present homology with their putative target genes outside the miRNA binding sites. This characteristic strongly suggests that certain young miRNA genes could be generated by inverted duplication of, at least, part of their target genes [10]. When such inverted repeat structures have the capacity to be expressed, the resultant hairpin products act as substrates for DCL complexes to generate a miRNA. Non-conserved miRNAs can often recognize a wide variety of transcripts [11], whereas conserved miRNAs principally target members of a single gene family that encode proteins involved in plant development, differentiation and stress responses [11, 12].

As it is the case with typical mRNAs, miRNA accumulation likely depends not only on their biosynthesis but also on their stability and degradation. By using a candidate-gene approach, Ramachandran and Chen [13] have identified one factor involved in miRNA degradation: the SMALL RNA DEGRADING NUCLEASES (SDNs). The SDN1 protein is able to degrade *in vitro* oligoribonucleotides of 17 to 27 nt into 8 to 9 nt. Moreover, a global increase of miRNA levels in *sdn* triple mutants strongly suggests that SDN proteins are involved in the miRNA pathway. The pleiotropic developmental phenotypes observed for these mutants demonstrate the crucial role of miRNA turn-over in plant development.

### B. siRNA Biogenesis

Plant siRNA populations proceed from endogenous or exogenous RNAs, which are converted into long dsRNA by RNA dependent RNA POLYMERASES (RDRs). These dsRNAs are cleaved by DCLs to generate siRNAs of different classes and sizes.* Arabidopsis* endogenous siRNAs contain three classes: (i) repeat associated siRNAs (rasiRNAs, showing the largest diversity), (ii) trans-acting siRNAs (tasiRNAs) and (iii) natural antisense transcript-derived siRNAs (nat-siRNAs). These different classes of small RNAs are generated by distinct pathways which include some overlapping functions. 

RasiRNAs are 24 nt long and derive from genome repetitive sequences. Their biogenesis requires the activity of RNA POL IV (formerly POLIVa, see below), RDR2, DCL3 and HEN1 [14]. RasiRNAs, which correspond to the most abundant small RNA category identified in all angiosperm species, guide sequence specific cytosine methylation of the homologous DNA, a process known as RNA dependent DNA Methylation (RdDM) or transcriptional gene silencing (TGS) [15]. Wierzbicki *et al.* [16] have recently renamed POLIVa and POLIVb as POLIV and POLV respectively and resolved part of the paradox of RNA-mediated chromatin silencing. Indeed, they showed that POLV, in association with the largest subunit NRPE1 (formerly NRPD1b), is responsible for the transcription of non coding RNAs that produce dsRNA substrates for DCL3. Then, rasiRNAs allow the induction of silencing by addition of epigenetic modifications such as DNA and/or histone methylation through the intervention of the RNA-induced Transcriptional Silencing complex (RITS) which encompass either AGO4 or AGO6 [17, 18]. More recently, the precise structure of these two plant specific RNA polymerases has been determined [19, 20]. *Arabidopsis* POLIV and POLV are composed of subunits that are paralogous or identical to the 12 subunits of POL II and these two enzymes differ at the level of four subunits.

The trans-acting siRNAs (tasiRNAs) are 21nt siRNAs that have a complex biogenesis involving the cleavage of non-coding transcripts, named TAS, by specific miRNAs. The cleaved transcript then enters a silencing pathway and is converted into a dsRNA by RDR6 assisted by SUPPRESSOR OF GENE SILENCING 3 (SGS3). The resulting dsRNA is further processed into phased 21 nt double-stranded siRNAs by DCL4 associated with DOUBLE–STRANDED RNA BINDING 4 (DRB4) in a sequential process initiated at the miRNA cleavage site. Some of these tasiRNAs are incorporated into RISC complexes, which encompass AGO1 or AGO7, to regulate their mRNA target genes *in trans*, like canonical miRNAs [21]. Noteworthy, these tasiRNAs play an important role in auxin responses and plant development through post-transcriptional regulation of their target transcription factors, AUXIN RESPONSE FACTOR 3 (ARF3) and ARF4 [22] or their actions against pentatricopeptide repeat proteins [23].

Finally, natural antisense siRNAs (nat-siRNAs) are produced from natural double-stranded RNAs generated by the concomitant expression of overlapping genes. The two nat-siRNAs currently known are involved in plant responses to salinity [24] and bacterial infection [25]. In both cases, one of the overlapping genes is constitutively expressed. The initiation phase involves DCL1 and DCL2 activities to generate the primary nat-siRNA from the overlapping region of the stress-induced and the constitutive mRNAs. This primary nat-siRNA directs the cleavage of transcripts from the constitutively expressed gene. In the amplification step, cleaved transcripts are converted into dsRNAs by RDR6, SGS3 and, curiously, POLIV [26]. Secondary nat-siRNAs are then processed by DCL1 from these dsRNAs and further promote the silencing of the constitutively expressed gene.

There are two classes of Natural Antisense Transcripts (NAT): *cis* –NAT and *trans*-NAT, which correspond to sense and antisense transcripts from the same or different genomic loci respectively. In *Arabidopsis*, Jin *et al*. [27] by using bioinformatics analyses, have identified small RNAs that matched at least one gene in 646 pairs out of 1008 predictions. Generally, the putative siRNA targets only one transcript of the given NAT pair, as previously found for salt and bacteria-induced nat-siRNAs [24, 25]. The same group has found 49 *cis*-NATs and 4769 *trans*-NATs by deep sequencing of control, drought and salt libraries in rice. Curiously, the majority of these small RNAs derived from only one of the genes in a NAT pair [28].

### C. Regulation of mRNA Targets

In animals, miRNAs inside RISC complexes, target transcripts at multiple sites in their 3’ untranslated regions (UTRs) through imperfect base pairing. The pairing region can sometimes be limited to 8 nucleotides (nt 2 to 9) in the 5’ part of the miRNA, so-called the miRNA seed. The interactions between the miRNA and its target(s) enable translation inhibition or progressive mRNA destruction. It has also been suggested that each animal miRNA has hundred of targets and that the strength of miRNA regulation is correlated to the number of molecules fixed on the 3’ UTR [29]. Thus, it has been proposed that cooperation between miRNAs during the regulation process may show a very strong sensibility to control mRNA translation as can be done by a rheostat [30]. By contrast, in plants, miRNA binding sites are generally found in the coding regions of the mRNA targets and show high complementarity with the miRNA. Their interaction essentially leads to the cleavage of the target, as it is the case for siRNAs. Hence, in plants, the main regulation seemed to be acting through mRNA decay. Due to the high level of homology between miRNAs and their targets, the number of putative targets for each miRNA is considerably limited. However, several recent studies revealed that these hypothesis are likely not correct. For plants, early studies on miR172 suggested that, although the miRNA encompasses highly complementary sequence with its APETALA 2 target, an inhibition of translation can arise [31]. Last year, Brodersen *et al.* [32], using genetic studies, clearly showed that several plant miRNAs, which present a high level of complementarity with their targets either in UTRs or in open reading frame, can strongly inhibit mRNA translation. Moreover, this capacity is genetically separable from target cleavage. Indeed, Brodersen and Voinnet revisit the principles of miRNA target recognition and mode of action in a nice recent review [33]. As mentioned there, “translation inhibition is commonly superimposed on slicing and may, under some circumstances, even dominate the outcome of miRNA-target interactions”. These authors also propose that, in plants, miRNA sliced sites might only correspond to “elite” sites and they do not exclude that large numbers of mRNAs could be targeted by imperfect pairing for translational inhibition, as it is the case in animals. These new results will certainly modify again our conception of gene regulation in plants and many current results may have to be revisited in this perspective.

## 3. IDENTIFICATION AND CHARACTERIZATION OF miRNA COMPLEXITY AND EXPRESSION

### A. miRNA Complexity

The complexity of small RNA populations has only been uncovered when high-throughput DNA sequencing was applied to this end. The best method to identify miRNAs is an alignment of the small RNA sequence read(s) against the reference genome or Expressed Sequence Tags (EST). This allows the identification of both hairpins corresponding to putative miRNA precursors and the potential miR* in the sequence collections. The identification of new miRNAs by this approach is thus limited to plant species where genomic information or large EST collections are available. In contrast, the identification of known miRNAs does not depend of the availability of the genome sequence. As the best quality data concerning conserved or new miRNAs derives from miRBase, we have only considered miRNAs registered in this database (release 12.0; September 2008, http://microrna.sanger.ac.uk/sequences/index. shtml) for the purpose of this review. MiRBase requires stringent criteria for miRNA annotations. For plants, previously published criteria [34] have been refined recently by Blake Meyers *et al.* [35]. Three criteria, which precise the excision of the duplex miRNA/miRNA* from the hairpin stem-loop, have been accepted for the miRNA definition: (1) miRNA/miRNA* must derive from a duplex with two 3’ nucleotides overhangs (signature of DCL processing), (2) very few mismatches are tolerated in the hairpin which generates the miRNA and (3) asymmetric bulges must be minimal in size and frequency in the miRNA/miRNA*. Moreover, the simultaneous identification of miRNA and miRNA* sequences, in the library, reinforces the prediction of a real miRNA.

#### Arabidopsis 

Initially, the identification of small RNAs in *Arabidopsis* were performed at low sequencing depth and thus only allowed the discovery of miRNAs that are the most abundant in different plant organs or in response to various stresses [36-41]. In contrast, the advent of high-throughput DNA sequencing such as Massively Parallel Signature Sequencing (MPSS) [42, 43], 454 pyrosequencing [44-46] and, more recently, SOLEXA technology permitted the discovery of less abundant small RNAs. Indeed, these latter sequencing methods produce very large number of reads for each sample examined. Currently, thanks to these new approaches, miRBase encompasses 187 Arabidopsis genes corresponding to 112 different miRNA families, including 73 that are specific from that species. Moreover, AthaMap [47], a database that integrates transcriptional and post-transcriptional data, has indexed 403 173 genomic perfect matches with the 109 590 MPSS of 17-mers identified by Lu *et al.* [42]. 

Together with the identification of new miRNAs, deep sequencing allows both to get an overview of small RNA diversity in one species but also theoretically to establish virtual expression profiles of small RNAs. For instance, in* Arabidopsis*, the global comparison of small RNA populations in different organs clearly demonstrated the preponderance of siRNA molecules in seedlings and flowers. Moreover, in all cases except for *rdr2* and *dcl3* mutants [45], siRNAs of 24 nt were the predominant class observed. This is consistent with the role of these proteins in the biogenesis of 24 nt rasiRNAs, which are concentrated across the pericentromeric regions of chromosomes [42]. On the other hand, miRNAs corresponded to the second most abundant class of small RNAs. A bimodal distribution of small RNAs with a minor peak at 21nt, comprising many redundant reads, and a major peak at 24 nt containing primarily unique or low abundant reads, has been generally observed in all libraries of angiosperm species.

The process generally used to identify new miRNAs consists in the bioinformatical search of RNA secondary structures in the genomic sequences that surround the small RNAs identified [36, 37]. Candidates for miRNA precursors correspond to hairpin structures without perfect repeated sequences and where, ideally, reads for the miRNA and the miRNA* could be identified. These candidates can often be identified in Expressed Sequence Tag (EST) libraries. It is important to note that, in all high-throughput sequencing projects published for *Arabidopsis*, many miRNAs identified corresponded only to one or very few reads and were encoded by a single locus. For example, the analysis of four libraries from seedlings, rosette leaves, flowers and siliques by Rajagopalan *et al.* [44], which theoretically covers a large part of the small RNA complexity, has revealed 221 676 reads (65%) only sequenced once among the 340,114 different small RNAs perfectly matching the genome. This phenomenon seems to be systematically found again in other studies [48, 49]. This suggests that our current knowledge of the small RNA diversity is far from complete even in *Arabidopsis*. However, deep sequencing has allowed the identification of either new miRNA genes or forms of mature miRNAs that differ only in some nt on the 3’ or 5’ ends from the conserved ones. Nearly identical miRNAs are generally grouped together into families. For plants with sequenced genomes, the list of paralog miRNA genes is easy to determine. At present, no miRNAs identified in the green alga *Chlamydomonas* have been found in *Arabidopsis* or other land plants [50]. In contrast, only some miRNA families identified in the moss *Physcomitrella patens* [51] are conserved in *Arabidopsis* and other angiosperms (Table **1**). This suggests the presence of early ancestors of miRNA genes, although a large divergence of miRNAs occurred after the appearance of *Chlamydomonas*.

The analyses of *Arabidopsis* small RNA high-throughput sequencing projects clearly suggest that further identification of miRNA genes in this species will probably necessitate to isolate small RNAs from specific cell populations, e.g. by using laser slice or cell sorting or by submitting plants to specific chemical effectors or stresses. By using laser-microdissection coupled to RT-PCR, Nogueira *et al.* [52] have been able to show that, in shoot apex, 390 miRNA precursors accumulate exclusively in leaf epidermis, whereas the mature miRNAs accumulate in sub-epidermal layers.

Until 2007, the prediction of miRNA targets in the plant kingdom was generally limited to mRNAs which present perfect or near perfect homologies with miRNAs [53]. Then, validations of putative targets were done by northern analyses or by using the 5’- rapid amplification of cDNA ends (5’-RACE) technique. This strategy, experimentally easy to realize when compared to the technologies used in animal kingdom, has unfortunately limited the number of putative targets to those yielding cleavage products. To detect a large number of plant targets cleaved by miRNAs, Addo-Quaye *et al.* [54] and German *et al*. [55] have developed a new approach using a modified 5’-RACE coupled with high-throughput DNA sequencing of 3’ mRNA cleavage products. This technique has been referred to as PARE (Parallel Analysis of RNA Ends). These authors have analyzed libraries from seedlings and inflorescences of** wild type and *xrn4* mutant. The *xrn4* mutant is deficient in the 5’ to 3’ exonuclease AtXRN4 which is involved in mRNA decay [56]. These experiments have allowed the confirmation of cleavage for many known Arabidopsis miRNA targets and the identification of several novel targets. However, as mentioned before, this ignores part of the mechanism of action of miRNAs in plants. It will be important now to determine how many of these miRNAs are also involved in the inhibition of protein synthesis and whether the nucleotide context or the location of the miRNA binding site in the target is involved in miRNA function as in animals [29].

#### Rice and Poaceae

The first 20 rice miRNA genes have been identified through their conservation with *Arabidopsis* [37, 38, 40]. Indeed, conservation of a small RNA in different species was a major criterion for the validation of a small RNA as a miRNA [34]. Before the advent of high-throughput DNA sequencing, several small RNA libraries were also constructed in this important crop, allowing the identification of abundantly expressed rice-specific miRNAs [57-59]. Nobuta *et al.* [60] have carried out a more thorough analysis of the rice transcriptome by using MPSS technology on mRNAs (22 libraries) and small RNAs (3 libraries) generating 46,000,000 and 1,900,000 signatures respectively. These sequences corresponded respectively to 250,000 and 280,000 non redundant sequences that matched the rice genome. As it was the case for *Arabidopsis*, the small RNA diversity from the inflorescence library was more complex than those of stems or seedling leaves. However, the absence of a complete set of biogenesis mutants in rice currently prevented to accurately distinguish between different pathways of small RNA biogenesis. Conserved miRNA families could be validated by MPSS data, as for many of them, both the miRNA and miRNA* were detected. In addition, many small RNAs, probably representing siRNAs, were found dispersed along chromosomes and not concentrated only in centromeric regions as observed in *Arabidopsis*. These data seem consistent with the fact that repetitive sequences are more dispersed in the rice genome. However, even if many of these small RNAs were derived from transposon or retrotransposon sequences, several also matched to intergenic regions with no repetitive sequence annotation. The rice genome could thus present regions specifically silenced by small RNAs involved in heterochromatin formation. This work has been complemented with the sequencing of three other small RNA libraries (seedling treated with ABA or the pathogenic fungi *Magnaporthe grisea*) [61]. Among the 4,400,000 MPSS signatures of 17 nt generated in the 6 different small RNA libraries, 57 % were detected only once, suggesting that this high depth was again not sufficient to saturate the population of rice small RNAs. In addition, nine unusual miRNAs have been identified, the so-called nat-miRNAs conserved only in monocots. These nat-miRNAs are generated from spliced natural antisense transcripts, which present a long double-stranded stem-loop RNA able to be processed by DCL enzymes. Although many similarities exist between nat-miRNAs and nat-siRNAs biogenesis where the dsRNA is generated by two overlapping transcripts [24], several evidences suggest that nat-miRNAs are true miRNAs. In addition to the presence of the structured dsRNA region, the accumulation of these nat-miRNAs is highly reduced in seedlings where DCL1 is repressed by RNA interference. Interestingly, all these nat-miRNAs target transcripts of MADS box genes.

Rice small RNAs have been recently analyzed using high-throughput DNA sequencing by several groups [48, 62, 63]. Rice-specific but also some monocot-specific miRNAs have been identified (Table **1**), suggesting either that some functions could be regulated through miRNAs in monocot and not in dicots or that certain miRNAs regulate monocot-specific functions. Interestingly, small RNAs from different stages of grain formation [48, 64, 65] have also been sequenced, as rice is both one of the most important food crops and one model species of the Poaceae family. These analyses revealed distinct populations of small RNAs in grains, including an abundant fraction of phased siRNAs of 21 nt. Moreover, around 50 miRNAs seemed specifically expressed during grain formation [48], suggesting that many developmental processes or metabolism pathways involved in grain maturation must be regulated by miRNAs.

Concerning other Poaceae, miRBase encompasses 32, 72 and 96 genes corresponding to 31, 16 and 17 miRNA families in wheat, sorghum and maize respectively. Only conserved gene families have been identified in maize and sorghum. Deep sequencing of small RNAs from the *mop 1-1* mutant, the maize ortholog of RDR2, has revealed a large proportion of 22 nt siRNAs, suggesting the existence, in maize, of a specific mechanism of heterochromatic siRNA production [66]. The *mop1-1* (loss of function) plants are not equivalent to the *rdr2* *Arabidopsis* mutants as the reduction of small RNA complexity is different: 80% for *Arabidopsis* and only 53 % for maize. Therefore, no new miRNAs could be identified by the analysis of the *mop1-1* small RNA population. This suggests that we must be careful to extrapolate, without verifications, results from *Arabidopsis* to other species. In contrast, 23 wheat-specific miRNAs identified by 454 sequencing are registered [67], although the large wheat genome is unknown. Yao *et al.* [67] used EST databases to predict hairpin structures that surround small RNA sequences. Moreover, this provided a direct evidence for the expression of miRNA precursors.

#### Other Species

In the gymnosperm *Pinus contorta,* 454 pyrosequencing allowed to identify 15 specific miRNA families that are registered in miRBase. Interestingly, the distribution of 21 nt and 24 nt small RNAs was inversed compared to *Arabidopsis* or rice [63], suggesting either that 21 nt small RNAs could replace heterochromatin siRNAs or that the number of miRNA genes is more important in gymnosperms. Future deep sequencing projects will answer whether this is a general characteristic of gymnosperms.

In poplar (*Populus trichocarpa),* 37 families of miRNAs are currently registered in miRBase coming from two low depth sequencing projects or bioinformatic analyses [68, 69]. However, results from pyrosequencing of small RNAs (bud and leaf tissues) from *P. balsamifera,* a *P. trichocarpa* close species,** have been recently published [70]. Curiously, as for the gymnosperm tree *Pinus contorta* [63]*,* the 21 nt small RNA class was more abundant than the 24 nt one in these small RNA libraries from perennial trees. It is interesting to note that, in poplar, most miRNA families are extended in size compared to *Arabidopsis* or rice; a characteristic also observed for the grapevine genes registered in miRBase. Additional analyses of miRNAs in perennial plants are required to correlate the size of gene families with perennial growth. Novel poplar miRNA families involved in specific tree development features may be identified when poplar depth small RNA libraries sequencing will be achieved.

In tomato, the identification of new miRNAs is difficult because the complete genome sequence is not yet available. MiRBase encompasses 30 miRNA genes corresponding to 17 different miRNAs families obtained by searching conserved homologs [71] or by 454 sequencing [72]. The latter approach was done by mapping small RNA sequences to Bacterial Artificial Chromosome (BAC) sequences. Only 4 tomato-specific new miRNAs could be identified, one targeting transcripts of a gene involved in fruit ripening.

In legumes, that are able to form nitrogen-fixing nodules in presence of rhizobia, the first small RNA libraries from root tissues were reported in soybean. Subramanian *et al.* [49] have constructed small RNA libraries from mock- or *Bradyrhizobium* *japonicum* inoculated roots (3h) to identify miRNAs putatively involved in early symbiotic interactions. Out of the 57 families registered in miRBase, 30 are new and certain were regulated during the first 12 hours after infection by rhizobia. Interestingly, the 21 nt small RNA class was more abundant than the 24 nt one. In addition, by using Genome Survey Sequence (GSS) and EST analyses, Zhang *et al.* [73] have identified a first plant antisense miRNAs. Thus, as described in animals [74, 75], some plant miRNA genes could be transcribed from both sense and antisense strands. Moreover, the GSS approach has allowed the observation of 5 miRNA gene clusters, 2 of them being absent in *Arabidopsis* or rice.

Currently, for the model legume *Medicago truncatula,* miRBase contains only 30 sequences of 10 conserved miRNA families. The recent results obtained from deep sequencing of aerial parts (stem and leaves) are not yet registered [76] but these authors have identified 26 novel miRNA candidates. For 8 of them, the miRNA* sequence could be detected.

### B. miRNA Expression

The knowledge of spatio-temporal miRNA expression patterns might provide clues about their functions. Mostly, Northern analyses are used to confirm the accumulation of mature form(s) of miRNAs. However, non-conserved miRNAs are often expressed at a very low level or, more likely, in specific cells or in a narrow time window, being therefore undetectable by this method. We have analysed *Medicago truncatula* libraries by deep sequencing [77] and found good correlation between sequence abundance in the libraries and northern analysis only for the most abundant conserved miRNAs. As mentioned in other studies [65, 70], the relative expression of miRNAs varied widely with respect to their proportion in sequenced libraries. More puzzling, recent results obtained from deep sequencing of three replicates of rice grain libraries indicated that only about 1,4 % or 6 % of unique sequences were common among three or two replicates respectively. This surprising result suggests that a bias during cloning processes, PCR amplification or bioinformatic analysis may exist in this approach. Theoretically, deep sequencing has clear advantages when compared to Northern or microarrays as it directly provides a quantification of each transcript without cross-hybridisation or signal detection problems. The number of MPSS signatures obtained from rice mRNAs were proportional to gene expression [60] and, in yeast, the so-called RNA-Seq which subjects fragmented complementary DNAs to high-throughput sequencing gave even more accurate results than qRT-PCR [78], allowing the observation of a complex transcriptome. These recent data in rice unveil questions about the limitation of deep sequencing to analyse small RNA abundance, as well as eventual sequencing errors imputable to pyrosequencing that could contribute to a bias during bioinformatics analyses of the data. This latter possibility will be more problematic. Other small RNA deep sequencings associated to robust statistical analyses are needed to answer those questions.

Alternative methods to examine miRNA expression patterns have been reported as miRNA chips [65, 69, 79] and qRT-PCR (quantitative RT-PCR) on mature miRNAs [80]. Xue *et al.* [65] have observed a good correlation between results obtained by chip and qRT-PCR for mature miRNAs of rice grains and, more interesting, even with abundant miRNAs identified by deep sequencing by Zhu *et al.* [48]. Results obtained by qRT-PCR and performed against pre-miRNA transcripts (and not against the mature miRNA) are more problematic as it cannot be excluded that, in some cases, pre-miRNAs are present in the cell without accumulation of mature miRNAs, as reported for miR390 in the shoot apex [52]. Hence, Northern analysis remains the more confident technique to monitor miRNA expression patterns. However, this method cannot distinguish between different members of a miRNA family. For that transgenic plant expressing reporter genes from putative promoter regions must be generated. By using this approach, it has been observed, for example, that the six loci of *Arabidopsis* miR395 are differentially regulated [81].

The expression patterns of some conserved miRNAs revealed a regulation by hormones [e.g. 82-85] or stresses [e.g. 69]. Some of the stress–responsive miRNA families are conserved among *Arabidopsis*, rice and poplar [e.g. 69, 86, 87] whereas others are species-specific. Moreover, in poplar, for example, conserved miRNAs may target different genes than in *Arabidopsis*, suggesting a functional diversification of these miRNAs in different species. To elucidate the modalities of miRNA gene regulation, Zhou *et al.* [88] have analyzed intergenic miRNA genes in animals (*Caenorhabditis elegans, Homo sapiens*) and plants (*Arabidopsis*, rice) and found that miRNA genes have similar promoter organization than protein-coding genes in both kingdoms, as their transcription is mainly RNA POLII dependent.

## 4. miRNA MECHANISMS OF ACTION DURING DEVELOPMENT

The important roles of miRNAs in plant development were demonstrated due to the pleiotropic phenotypes of mutants, such as *dcl1,* associated to the miRNA biogenesis pathway [89]. Strong alleles of *dcl1,* one of the major enzymes of miRNA biogenesis, are embryo lethal whereas hypomorphic ones showed diverse developmental anomalies. The different mechanisms of miRNA action previously described (part II, section C) have been observed for several mRNA targets during plant development. 

### A. Major miRNA Regulations Occur During Shoot Organogenesis

Direct impact of specific miRNAs on development have been initially observed by using transgenic plants that overexpress either a miRNA or miRNA-resistant mRNA targets of specific miRNAs [e.g. 90-92]. In addition, mutations affecting the miRNA-targeting site of key regulatory mRNAs also led to drastic developmental phenotypes by perturbation of the post-transcriptional regulation of this specific target. Currently, developmental roles for plant miRNAs are mainly linked to mRNA target stability. A first example was the developmental regulation by miR319 of the TEOSINTE BRANCHED1 CYCLOIDEA AND PCF FAMILY (TCP2) mRNA: cleavage of this transcript is essential to control leaf morphogenesis [93]. Furthermore, the action of miR165/166 spatially restricts the expression of the PHABULOSA gene family during the establishment of leaf polarity. Indeed, miR165/166 and its target accumulate in adaxial and abaxial domains of the leaf respectively [94, 95]. In contrast, miR172 controls, at least partly, the temporal transition from the vegetative to the flowering phase by translation inhibition of an APETALA2 transcription factor [96]. On the other hand, miR164 regulates the accumulation level of CUP-SHAPEP-COTYLEDON (CUC) transcripts in boundary domains between floral primordial and floral meristem through a rheostat mechanism [97]. Several related examples of miRNA-mediated regulation during shoot organogenesis have been proposed [98].

### B. miRNA Regulation in Root Development

The knowledge on miRNA action in root development is succinct as basically very few small RNA libraries of root tissues have been analysed. As mentioned above, deep sequencing of small RNAs from root tissues was reported in soybean, a legume crop [49]. Nevertheless, although new miRNA candidates were discovered, their involvement in root growth or development was not studied. In contrast, the action for miRNAs that target genes linked to root developmental processes have been analysed in *Arabidopsis*. The regulation of another member of the NAM/ATAF/CUC (NAC) family by miR164 was evidenced using miR164-resistant NAC1 targets that increased the number of lateral roots [82]. The accumulation of miR164 is regulated by auxin, a key hormone controlling all steps of lateral root formation from initiation to emergence. In *Arabidopsis*, several miRNAs are involved in auxin signalling through their action on AUXIN RESPONSE FACTORS (ARFs). MiR160 targets ARF10, ARF16 and ARF17 transcripts [99], whereas transcripts encoding ARF3 and ARF4 proteins are recognized by tasiRNAs derived from the cleavage of TAS3 transcripts by miR390 [100]. The regulation of ARF10 and ARF16 by auxin and miR160 is involved in root cap development [99] whereas miR-resistant versions of these targets affect adventitious and lateral root formation [84].

Through their regulatory roles in mineral nutrition or stress responses, miRNAs may be also involved in the control of root architecture and its adaptation to environment. Indeed, miR399, miR395 and miR398 are induced by low availability of phosphorus [101], sulphur [102] and copper [103] respectively, mineral nutriments whose availability strongly affects root architecture in *Arabidopsis* [104]. MiR399, which is up-regulated in low phosphate medium, targets PHO2 transcripts and is a key element for regulation of phosphate homeostasis at plant level [105]. This miRNA is expressed in companion cells of the phloem and can be transported from shoot to root suggesting that it may participate in long-distance signalling in the plant [105, 106, 107]. This phenomenon was also observed for miR395 [101]. In addition, using miRNA microarrays that encompass about 700 miRNAs from different plant species, Zhang *et al.* [108] and Ding *et al.* [109] have identified certain modifications of maize root miRNA expression patterns consecutive to root submergence or salt treatment. Those miRNAs, such as miR164 and miR166, target transcription factors involved in root development.

During symbiotic interactions in legumes, a new organ is derived from the primary root, the root nodule. This organogenesis requires the coordinated expression of several transcription factors [110] including *MtHAP2.1,* a transcription factor expressed only in nodule meristem. This transcription factor is rapidly down-regulated once differentiation starts through the action of miR169 [111]. In addition, MIR166 has been shown to post-transcriptionally regulate a family of HD-Zip III (homeodomain-leucine zipper class III) transcription factors expressed in vascular tissues and associated with nodule formation. Overexpression of miR166 down-regulates the expression of these genes and, concomitantly, affects root vascular tissues patterning in *M. truncatula* [112]. Both initiation of symbiotic nodules and lateral roots are impaired in these miR166 over-expressing roots, possibly because of the mispatterning of vascular bundles. As miR166 also regulates vascular development in *Arabidopsis* shoots [113], this miRNA may have a general role in regulating formation of secondary organs in plants. 

Hence, miRNAs contribute to the spatial and temporal regulation of transcription factors in roots during de-differentiation and re-differentiation processes occurring in the tissues that lead to the formation of new meristems. In addition, other miRNA-target classes may be involved in the regulation of root responses to the environment. The identification of new miRNAs and their integration into the regulatory networks of root growth and development may be influential to adapt the root architecture to a specific soil environment.

## Figures and Tables

**Table 1:** The Conserved miRNA Families in Plants

Family	Eudicots						Monocots				Other Embryophyta		
	Ath	Ptc	Vvi	Gma	Mtr	Sly	Osa	Zma	Tae	Sbi	Ppt	Smo	Pta
156	X	X	X	X	X	X	X	X		X	X	X	X
159	X	X	X	X		X	X	X	X	X		X	X
160	X	X	X	X	X	X	X	X	X	X	X	X	
162	X	X	X	X	X	X	X	X					
164	X	X	X	X			X	X	X	X			
166	X	X	X	X	X	X	X	X		X	X	X	X
167	X	X	X	X		X	X	X	X	X	X		
168	X	X	X	X			X	X		X			
169	X	X	X	X	X	X	X	X		X			
171	X	X	X	X	X	X	X	X	X	X	X	X	X
172	X	X	X	X		X	X	X		X			
319	X	X	X	X	X	X	X	X		X	X	X	X
390	X	X	X	X			X				X		X
393	X	X	X	X	X		X	X		X			
394	X	X	X				X	X		X			
395	X	X	X		X	X	X	X		X	X		
396	X	X	X	X			X	X		X		X	X
397	X	X	X			X	X						
398	X	X	X	X			X						X
399	X	X	X		X	X	X	X	X	X			
408	X	X	X				X	X	X		X	X	X
413	X						X						
414	X						X				X		
415	X						X						
416	X						X						
417	X						X						
418	X						X						
419	X						X				X		
420	X						X						
426	X						X						
530		X					X						
535			X				X				X		
827	X	X					X						
													
482		X	X	X									X
403	X	X	X										
472	X	X											
477		X	X								X		
479		X	X										
783	X												X
828	X		X										
845	X		X										
													
444							X		X				
529							X				X		
													
536											X	X	
specific families	73	14	0	30	0	4	91	0	23	0	85	36	15

MiRNA families registered in at least 2 (among 13) plant species in the miRNA database miRBase v12 (http://microrna.sanger.ac.uk/sequences/index.shtml) are listed. Crosses (X) indicate that the family is present in the corresponding species. *Arabidopsis thaliana*: Ath, *Populus trichocarpa*: Ptc, *Vitis vinifera*: Vvi, *Glycine max*: Gma, *Medicago truncatula*: Mtr, *Solanum lycopersicon*: Sly, *Oryza sativa*: Osa, *Zea mays*: Zma, *Triticum aestivum*: Tae, *Sorghum bicolor*: Sbi, *Physcomitrella patens*: Ppt, *Selaginella moellendorffii*: Smo and *Pinus taeda*: Pta.
